# Reference frames in virtual spatial navigation are viewpoint dependent

**DOI:** 10.3389/fnhum.2014.00646

**Published:** 2014-09-09

**Authors:** Ágoston Török, T. Peter Nguyen, Orsolya Kolozsvári, Robert J. Buchanan, Zoltan Nadasdy

**Affiliations:** ^1^Doctoral School of Psychology, Eötvös Loránd UniversityBudapest, Hungary; ^2^Department of Cognitive Psychology, Eötvös Loránd UniversityBudapest, Hungary; ^3^Research Centre for Natural Sciences, Institute of Cognitive Neuroscience and Psychology, Hungarian Academy of SciencesBudapest, Hungary; ^4^Department of Psychology, University of Texas at AustinAustin, TX, USA; ^5^Division of Neurosurgery, Seton Brain and Spine InstituteAustin, TX, USA; ^6^Department of Psychiatry, UT Southwestern Medical CenterDallas, TX, USA; ^7^NeuroTexas Institute, St. David's HealthcareAustin, TX, USA

**Keywords:** survey knowledge, navigation, perspective taking, point of view, egocentric, allocentric, tablet pc, virtual reality

## Abstract

Spatial navigation in the mammalian brain relies on a cognitive map of the environment. Such cognitive maps enable us, for example, to take the optimal route from a given location to a known target. The formation of these maps is naturally influenced by our perception of the environment, meaning it is dependent on factors such as our viewpoint and choice of reference frame. Yet, it is unknown how these factors influence the construction of cognitive maps. Here, we evaluated how various combinations of viewpoints and reference frames affect subjects' performance when they navigated in a bounded virtual environment without landmarks. We measured both their path length and time efficiency and found that (1) ground perspective was associated with egocentric frame of reference, (2) aerial perspective was associated with allocentric frame of reference, (3) there was no appreciable performance difference between first and third person egocentric viewing positions and (4) while none of these effects were dependent on gender, males tended to perform better in general. Our study provides evidence that there are inherent associations between visual perspectives and cognitive reference frames. This result has implications about the mechanisms of path integration in the human brain and may also inspire designs of virtual reality applications. Lastly, we demonstrated the effective use of a tablet PC and spatial navigation tasks for studying spatial and cognitive aspects of human memory.

## Introduction

Following Tolman's seminal work, it has been widely assumed that mammalian spatial navigation relies on cognitive maps (Tolman, 1948). However, how these maps are acquired is largely unknown. Cognitive maps are thought to be allocentric, meaning their representations of the environment are independent of the individual. Yet, the sensory experience that usually leads to the construction of these maps is dependent on the individual's egocentric experience (Siegel and White, 1975). Continuous spatial information can be inferred from optic flow in a number of ways, from first person to an infinite number of external virtual “camera” positions, even if those camera positions are disjoined from the object the participant needs to navigate. Amongst these innumerable options, the type of sensory projection most effective at supporting spatial navigation is still uncertain (McCormick et al., 1998). This question is not only a matter choosing the effective “camera angle,” but also the effective cognitive frame of reference.

Theoretically, we distinguish between two fundamentally different types of reference frames: egocentric and allocentric (Klatzky, 1998). While egocentric navigation aligns the coordinate system relative to the agent (e.g., to the “right” or “left”), allocentric navigation aligns the coordinate system relative to the environment (e.g., “North” or “*next to …*”). This duality of reference frames is reflected by the differential anatomical localization of reference frames. During physical navigation, our visual sensory experience of the environment is predominantly egocentric—the LGN and the V1-V2 areas of the visual cortex define space in retinotopic coordinates. Neuronal representations of space along the dorsal stream (Goodale and Milner, 1992), become progressively independent from the retinal coordinates and increasingly body centered. For example, while the lateral intraparietal (LIP) areas represent information in retinotopic coordinates (Kusunoki and Goldberg, 2003), the ventral intraparietal sulcus (VIP) encodes information in head centered coordinate systems (Avillac et al., 2005), and anterior intraparietal sulcus (AIP) encodes according to body-centered coordinate systems (Fogassi and Luppino, 2005). In general, the parieto-occipital areas represent the egocentric realm of spatial sensory processing.

In contrast, the mesio-temporal cortical structures, including the hippocampus and entorhinal cortex, encode space in allocentric coordinates. In the entorhinal cortex and hippocampus, where the dorsal and ventral pathways converge (Felleman and Van Essen, 1991), the majority of cells obtain spatial specificity by responding to spatial locations of the agent relative to external landmarks. The most notable among these cells are place cells in the hippocampus and grid cells in the entorhinal cortex (O'Keefe and Nadel, 1978; Ekstrom et al., 2003; Hafting et al., 2005).

Studies on the formation of spatial representations in the brain distinguished three stages (Linde and Labov, 1975; Siegel and White, 1975). First, landmarks are identified (landmark knowledge), then a place-action representation map is created (route knowledge), and finally a configurational map of the environment is constructed (survey knowledge). These stages of spatial knowledge are typical for direct navigation. However, we often explore space in a qualitatively different way: by using maps. Whereas first person navigation is primarily egocentric, maps are the archetype of allocentric representation. Zhang et al. (2012) in their neuroimaging study compared the engagement of brain areas between two conditions set up prior to the spatial task: when participants learned the spatial layout by navigating through it firsthand vs. by viewing a map of the environment. They found greater activation in the parahippocampal and the retrosplenial cortex after direct navigation, possibly reflecting the conversion from egocentric to allocentric representations. After map learning, the inferior frontal gyrus showed greater activation. The change is, according to the authors, associated with the conversion from allocentric to egocentric coordinates. Other studies also found that map-like perspectives lead to somewhat different activations in the spatial processing networks (Shelton and Gabrieli, 2002; Zaehle et al., 2007). These studies raise the question: what is the key difference between presentations of the same spatial information that leads to navigation according to an allocentric reference frame in one scenario, and according to an egocentric reference frame in another? More specifically, what is the critical factor that determines the choice of reference frame during spatial navigation? Based on these earlier experiments, it is expected that first person points of view favor an egocentric reference frame, while map-like aerial presentations favor allocentric reference frames. It is not clear how 3rd person ground level perspectives, lying somewhere between first person and map-like perspectives, affect navigation performance. In order to answer this question we had to remove confounding factors from our paradigm that affected the interpretation of earlier studies.

Firstly, maps convey spatial information differently from direct first person navigation in a number of ways. Most obviously, maps employ a different perspective, taking an aerial point of view instead of a ground level perspective (Török, 1993; Snyder, 1997). Maps also offer a bigger overview of the environment and hence easier recognition of landmarks and borders. Moreover, since maps typically show the boundary of space, they provide a reliable reference for the avatar's position (Brunyé et al., 2012). All these factors could potentially play a role in biasing performance between map-like vs. first person views in navigation. In their study, Barra et al. (2012) found that a slanted perspective, which gave more overview on the environment, led to better performance in a shortcut finding task. However, they manipulated not just the size of overview but the camera position as well. Distance perception is also affected by the field of view (Alfano and Michel, 1990; Kelly et al., 2013). Although it is not possible to balance the field of view between ground-level and aerial perspectives, it is possible to balance the average visible area. If the field of view (FOV) from a fixed aerial perspective is constant, then the effective FOV for ground-level perspective should be controlled too. In their study, Shelton and Pippitt (2007) followed a similar approach, though in their task the navigable area contained several occluders thus rendering the comparison across different visibility conditions ambiguous. When comparing navigation performances across different perspectives, bounded but open areas with equally visible portions in every viewpoint are preferred in order to avoid biases derived from different FOVs.

Secondly, although maps are typically allocentric, users often prefer to turn the map according to their current heading, thereby using them egocentrically. This suggests that the reference frame of maps may depend on additional factors. For example, Wickens and colleagues found that pilots landed in simulated environments better when the 3D-map was locked to the airplane's orientation as opposed to in environments where the view was locked to the north-south axis (Wickens et al., 1996; see also Eley, 1988). However, other results show that fixed orientation aerial perspectives lead to better configurational knowledge due to the consistency in global orientation over time (Aretz, 1991; McCormick et al., 1998). Furthermore, results derived from three-dimensional flight simulator data may not directly generalize to two-dimensional spatial navigation.

Thirdly, the flight simulator experiments introduced another confounding factor: the view of the airplane from an outside point of view. This is analogous to the configuration of a visible avatar, commonly applied in many computer games as well as the stereotypical representation of the protagonist we identify with in films. The precise effect of a visible avatar on learning navigation, even when it is aligned with the subject's point of view, is unknown. Studies demonstrated that the sense of actual presence in a virtual environment is weakened when the self-avatar was viewed from a 3rd person point of view (Lenggenhager et al., 2007; Slater et al., 2010). To test whether the outside view on the avatar has an intermediate effect relative to the 1st person and bird-eye points of view, we included the 3rd person point of view to our design to help decipher the relationship between reference frames and camera views.

In summary, answering the question of whether certain combination of perspective and camera movement is preferentially associated with egocentric vs. allocentric frame requires combining three different camera views (map-like, 3rd person and 1st person views) and two reference frames (egocentric and allocentric); a paradigm that has not been applied.

We implemented the task as a computer game in which we independently varied the camera views (ground-level vs. bird-eye perspectives) and the orientation of the camera (follow avatar's heading vs. always north). Like in the Shelton and Pippitt (2007) study, we balanced the average visible navigable area between perspective conditions. The dependent variables were the navigation time and navigation path length relative to the optimal value for each.

We further introduced a few important constraints: the environment was bounded by limiting the navigable area with walls; no landmark cues other than the walls were available; and the compartment had a square geometry with visually equivalent corners, making it a less reliable orientation cue (i.e., the corners were rotationally symmetric, see Pecchia and Vallortigara, 2012). In order to compare the accuracy of the cognitive maps stored in memory as opposed to comparing navigation accuracy relative to visible targets, we rendered the targets invisible.

We also provided an avatar during ground-level and aerial navigation so participants were able to see themselves from an outside perspective. Because natural ground-level navigation takes a 1st person perspective, we used this as a baseline condition. We hypothesized that 3rd person navigation in an egocentric reference frame would not produce differing navigation performance when compared to the natural 1st person navigator's perspective. Additionally, we modeled the avatar as a human as opposed to representation by a cursor, as was done in earlier experiments (Barra et al., 2012). Because both the visible area and the presence of an avatar were balanced across the viewing conditions, differences in navigation accuracy were only attributable to an inherent association between perspective and frame of reference. In our experiment we dissociated the two factors (view and camera movement) by alternating the reference frames between egocentric and allocentric coordinate systems while also cycling the point of view between first person, third person (above and behind the avatar) and an aerial view. We hypothesized that the ground level perspective was associated with an egocentric frame of reference in navigation whereas an aerial perspective would evoke the use of an allocentric frame of reference.

## Methods

### Participants

Fifty participants (25 female) took part in the experiment. Their age ranged from 18 to 32 years (mean: 21.93). Forty-six were right handed. All participants were university students. Prior to the experiment, it was verified that the participants could see and hear the stimuli well. Participants gave written informed consent and received course bonus points for participating. The study was approved by the research ethical board of the ELTE University and met the principles of the Declaration of Helsinki.

### Apparatus and stimuli

The virtual reality game was programmed in Unity 3D (Unity 4, www.unity3d.com). The game was played on an Asus TF 201 and an Asus TF 301 lightweight tablet PC (NVIDIA® Tegra® 3 Quad Core CPU, 1Gb DDR3 RAM, Android™ 4.x). The devices had a 10.1-inch capacitive multi-touch display with a resolution of 1280 × 800 pixels. The tablet was chosen as a stimulus presentation interface because we use the same virtual reality paradigm for testing epileptic patients in clinical settings where the portability, the lightness of device, and the ease of control are primary constraints.

The paradigm was a custom game called “Send Them Back Home.” The goal of the game was to collect space aliens holding a colored briefcase and to carry the aliens to their spaceships of matching color. The game's scenario was similar to the Yellow Cab game developed by Caplan et al. (2003). Like in Yellow Cab, the target objects (aliens) were placed quasi-randomly while the two goal places (spaceships) were at fixed locations, so the task involved beacon aiming during the searching phase and path integration (dead-reckoning) during the delivery phase of the experiment. The target objects were 1.5 unit tall alien figures that carried either a yellow or blue briefcase. The two spaceships were simple 3.5 unit diameter and 1.5 unit tall flying saucer-like objects with either a yellow or blue body. To force reliance on memory and external spatial cues rather than the visible spaceship, the spaceship targets were visible only at the beginning of the game. That is, after the first alien delivery to each spaceship, the spaceships became invisible except when the avatar was within a 6-unit radius of a ship. Participants were told that the spaceships were using a cloaking machine to hide their location. The virtual environment was a large square-shaped yard enclosed by brick walls. The sky was uniform blue and the ground was covered with a grass texture. The size of the environment was 80 × 80 unit, and the wall was 5 unit tall.

**Figure 1:**
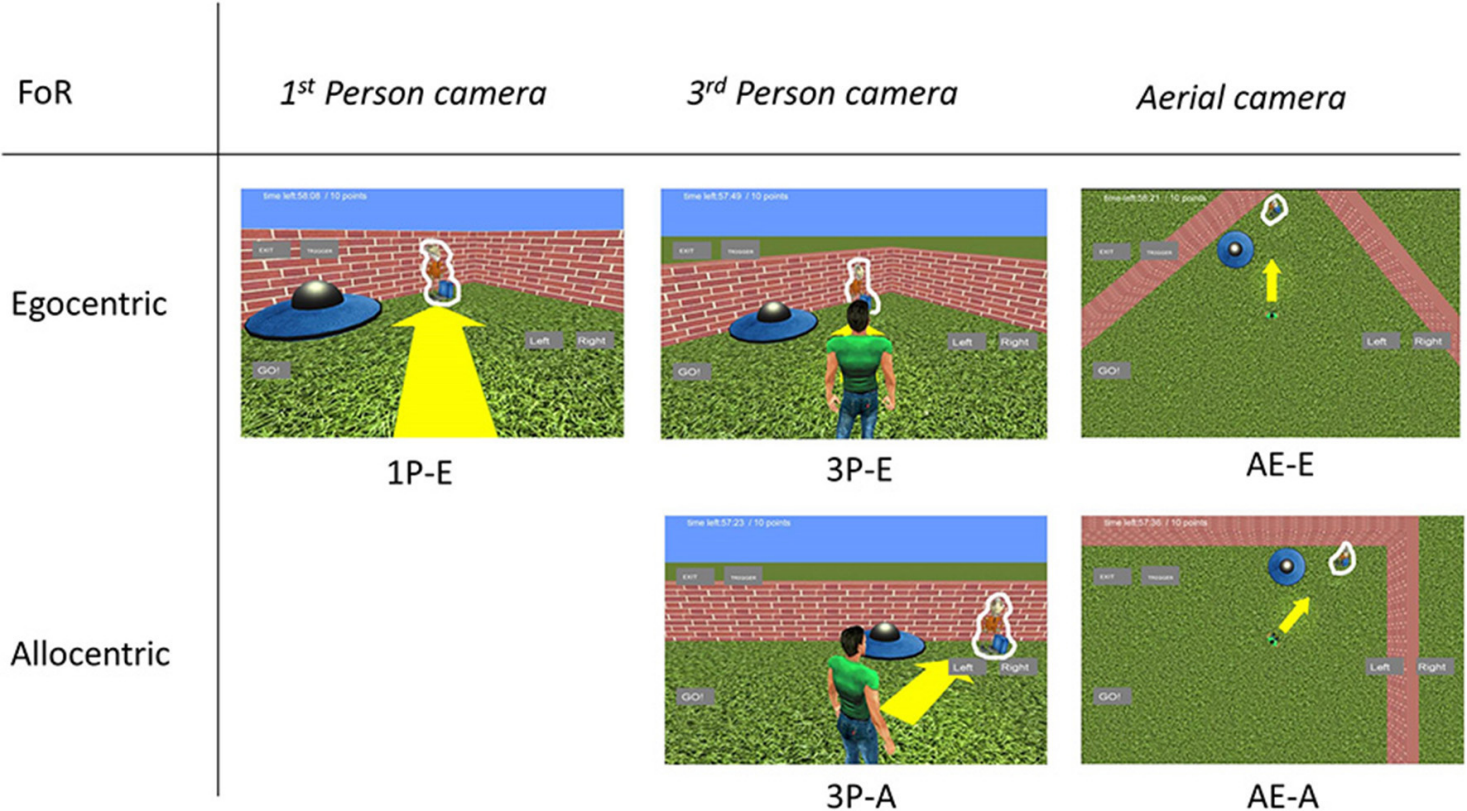
**Sample views from the five camera modes used**. We used three different camera modes: 1st Person camera was a ground level point of view; 3rd Person camera was a camera at a fixed 3.5 unit distance relative to the avatar and looked down from a 20° slanted perspective; the Aerial point of view was a map like perspective, 16.5 m above the field. For the last two the camera orientation was fixed relative to either the avatar or the environment. The arrow is visible and the alien figures are outlined with a white contour only for presentation purposes.

We tested five different camera setups created from combinations of different views and orientation modes (see Figure 1) in a within-subject design. The views consisted of a 1st person view (eye height 2 unit), 3rd person view (3.5 unit behind the avatar, 4.5 unit above the ground, and slanted 20° downward) and an aerial view (birds-eye view from 16 unit above). The orientation modes were egocentric (camera turned to follow avatar's heading) and allocentric (permanent always-north camera orientation). Excluding the impossible 1st person-allocentric combination, this resulted in: (1) a 1st person egocentric camera mode (1P-E) (2) a 3rd person egocentric camera mode (3P-E) (3) a 3rd person allocentric camera mode (3P-A) (4) an aerial egocentric camera mode (AE-E), and (5) an aerial allocentric camera mode (AE-A). The average field of view was balanced between camera modes to ~910 m^2^ (3P = 1P = ~908 m^2^; AE = ~912 m^2^).

Motion was controlled by pressing an on-screen “GO” button with the left thumb and a “LEFT,” or “RIGHT” button with the right thumb. Simultaneous touch of the “GO” and arrow buttons allowed for continuous steering in the virtual space. The speed of the participant was 5 unit/s, and step sounds were played during forward movement. Turning speed was 80°/s. The player's virtual trajectory, including heading, was logged every 50 ms. This trajectory information was saved to the tablet's internal memory in a text file along with the coordinates of alien placements.

### Procedure

Participants were sitting in front of a table holding the tablet in their hands. Prior to the experiment, they were told that they had to search for misplaced aliens and return them to their spaceships. They were instructed to deliver as many aliens as they could during the game. They were also told that after each delivery the camera mode would switch, but that the spaceships would not change their position. Lastly, they were warned to make note of spaceship locations at beginning of the task because after the first delivery to each spaceship, they would activate their cloaking mechanism.

**Figure 2:**
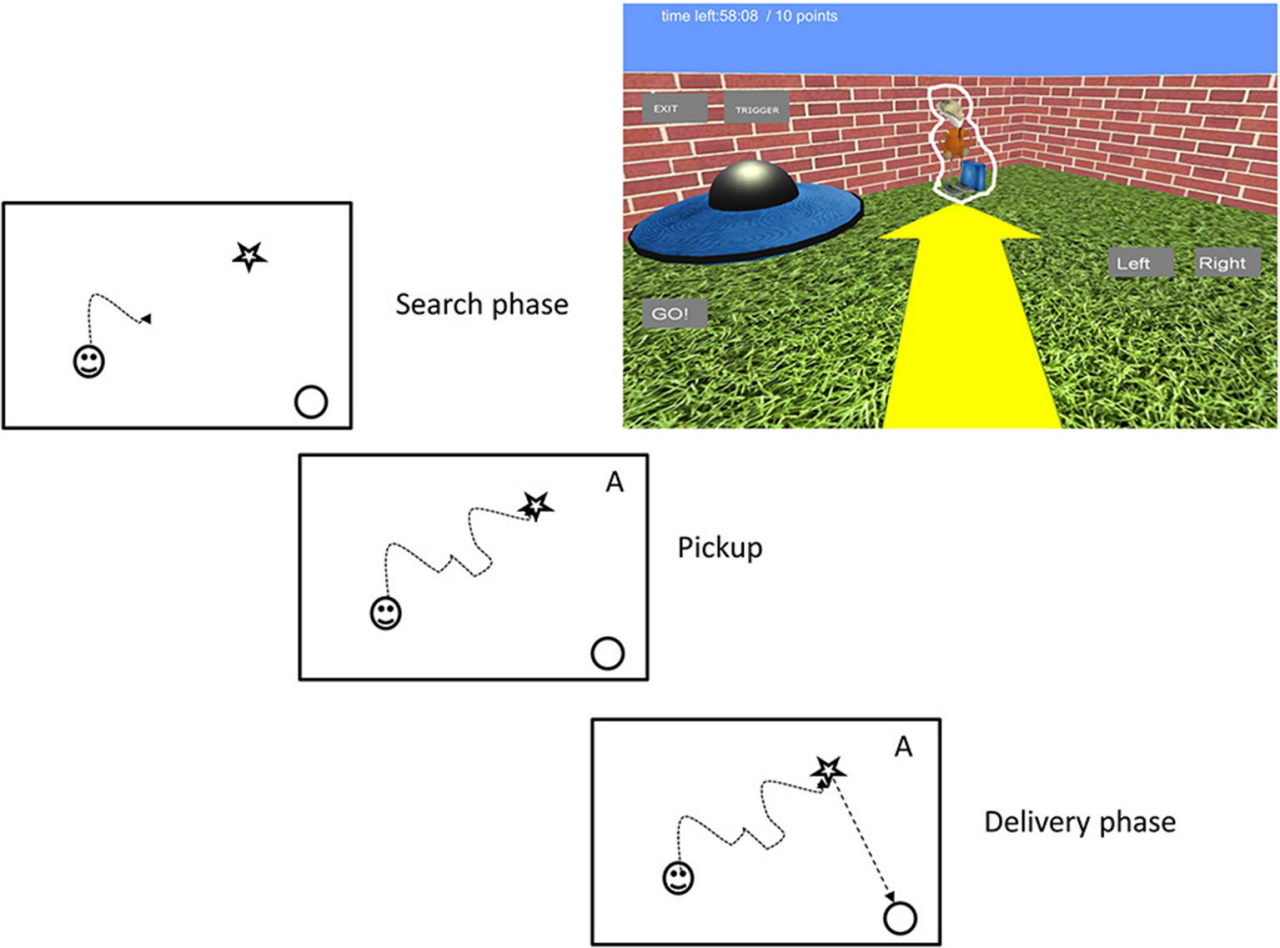
**The virtual reality task. In the search phase participants looked for a space alien**. They picked it up by running through it. Carrying of an alien was indicated by an alien image on the top right corner of the screen (symbolized by an “A” here for simplicity) to indicate the next phase. In the delivery phase they carried the alien to its spaceship. Upon contact with the correct spaceship a new alien appeared. The game was controlled by onscreen GO, LEFT, and RIGHT buttons. For illustration purposes we outlined the alien figure with a white contour.

Each trial started with an alien in the environment. The participants searched for the alien and picked it up by walking over it (see Figure 2). When they picked up the alien a small alien figure appeared in the top right corner with text indicating the target spaceship's color. At the same time the alien gave audio instructions about the next task by saying “Now take me to my spaceship.” Delivery of the alien to the appropriate spaceship was signaled by the alien saying “thank you very much” and rewarded with 1 point in the game score. A new alien was then placed in the map. The camera modes alternated in a random order after each delivery, but without returning to a previous camera mode until all five of the possible modes had been cycled through. This means that each subject was tested under all five viewing conditions that enabled us to compare performances within subjects. To maximize the subject's map coverage during play, aliens were spawned at 1 of 28 preset locations, selected randomly without resampling until necessary.

Each experiment lasted for 30 min. Before the experiment, subjects practiced the touchscreen controls in a training environment.

### Data analyses

Differences in performance due to camera mode were analyzed by comparing the player's efficiency on the alien delivery portion of the task (i.e., only when returning an alien to its spaceship). Performance was scored both in terms of route efficiency and time efficiency. The former was defined as a performance measure called route performance and equaled the percentage of the player's actual trajectory (Δ*d*) to the shortest possible route (*d*_*ideal*_). Since there were no obstacles, *d*_*ideal*_ was taken as the straight-line distance between the alien pick-up point and the target spaceship:

$$d_{ideal\;=}\sqrt{x^{2}+y^{2}}$$

Time efficiency for alien deliveries was quantified as a time performance statistic equal to the percentage of observed delivery time (Δ*t*) from the shortest possible delivery time (*t*_*ideal*_). The ideal phase completion time was calculated by the equation below, where *x* and *y* are the coordinates for the absolute distance, α is the minimum angle needed to turn from the current heading to the spaceship, *v*_*forw*_ is the speed of forward motion and *v*_*turn*_ is the speed of turning (both speeds were constant).

$$t_{ideal}=\sqrt{{\left(\frac{\sqrt{x^{2}+y^{2}}}{v_{forw}}\right)}^{2}+{\left(\frac{\alpha }{v_{turn}}\right)}^{2}}$$

Although path length and path time are closely related, they are not always proportional, except when the avatar is continuously moving toward the target in a straight line. All other times, either when turning without moving or when the turning and advancing create a curved trajectory, which may be optimal in time but suboptimal in path length, the two are disproportionate. Therefore, the two parameters are highly correlated but not identical. Nevertheless, we had no basis to exclude either parameter and computed both.

Because we were interested in the delivery phases when the participant had to rely on their spatial memory (path integration), we only analyzed the trials where the destination spaceship was not visible at the time of pickup (i.e., *d*_*ideal*_ > non-cloaking radius). Following this criterion, on average we excluded 2.02 delivery trials (min: 0; max: 4). For the same reason, we excluded all first visits to each spaceship, as the cloaking mechanism only activated afterwards. Furthermore, in some trials participants did not simply take suboptimal routes but completely lost track of where to go. Because these trials were not artifacts *per se*, we decided not to exclude them. Instead, we winsorized the upper 5% of all data (0–7 data points for every person; mean: 2.90). Therefore, we did not analyze the extreme values, yet were able to include those trials in analysis. Regardless, trimming instead of winsorization did not change the main results.

## Results

### Overall performance

We were interested in how different points of view and frames of reference affect navigation performance during alien delivery. Although the average field of view was balanced across viewing conditions, the period when players searched for aliens was excluded from our analysis because this task favors the 1st person and 3rd person egocentric camera modes. These modes allow the player to visually search the map with one quick 360° rotation of the avatar. Meanwhile, the aerial camera mode, which reveals only 912 m^2^ of the 80 × 80 m environment, requires the player to search for aliens by physically roaming the environment. This disparity was not present during the alien delivery phase because the target spaceships were invisible and permanent in location. We therefore analyzed performance in only the delivery phases. Across the 30-min trial, participants collected 57.34 (*SD*= 9.08) aliens on average. Of note, we also found that male subjects tended to perform better than female subjects [60.24 (*SD* = 9.00) > 54.4 (*SD* = 8.35); *t*_(1, 44)_ = 2.36; *p* = 0.022].

Since each participant was tested under all five viewing conditions but analyzed according to route length and time performance, we applied a within-subjects repeated measure ANOVA design separately for the route length and for the time performance variables. We present these results accordingly.

### Optimality of route length performance

**Figure 3:**
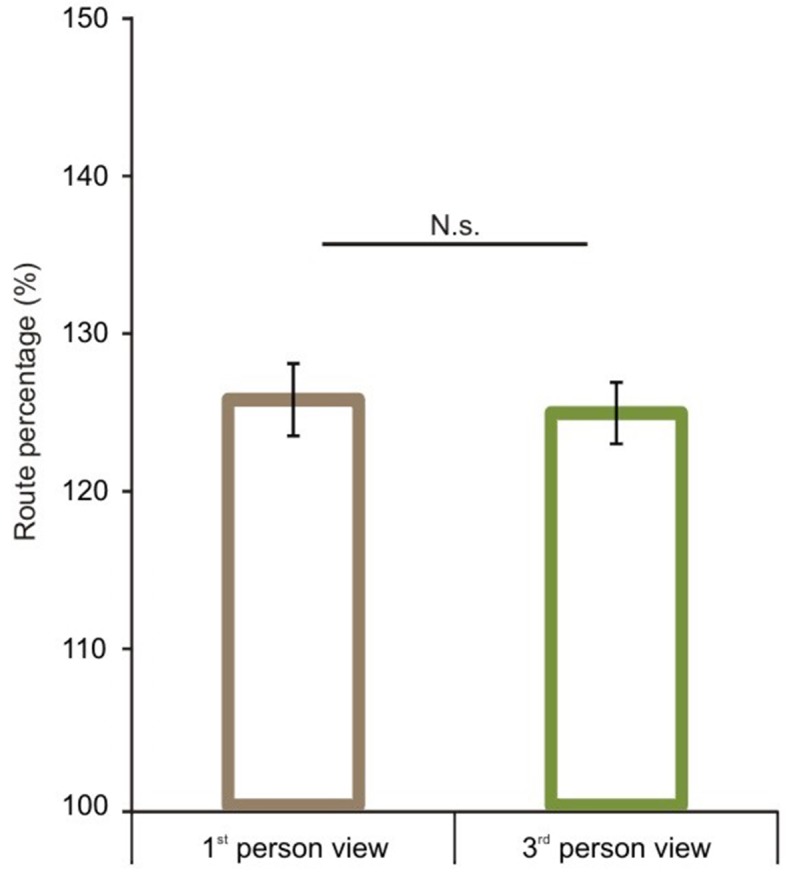
**Route performance scores in the 1st and 3rd person viewing conditions**. No difference was found between 1st person and 3rd person views when both represented egocentric frames of reference. Vertical bars denote standard errors. n.s., not significant.

**Figure 4:**
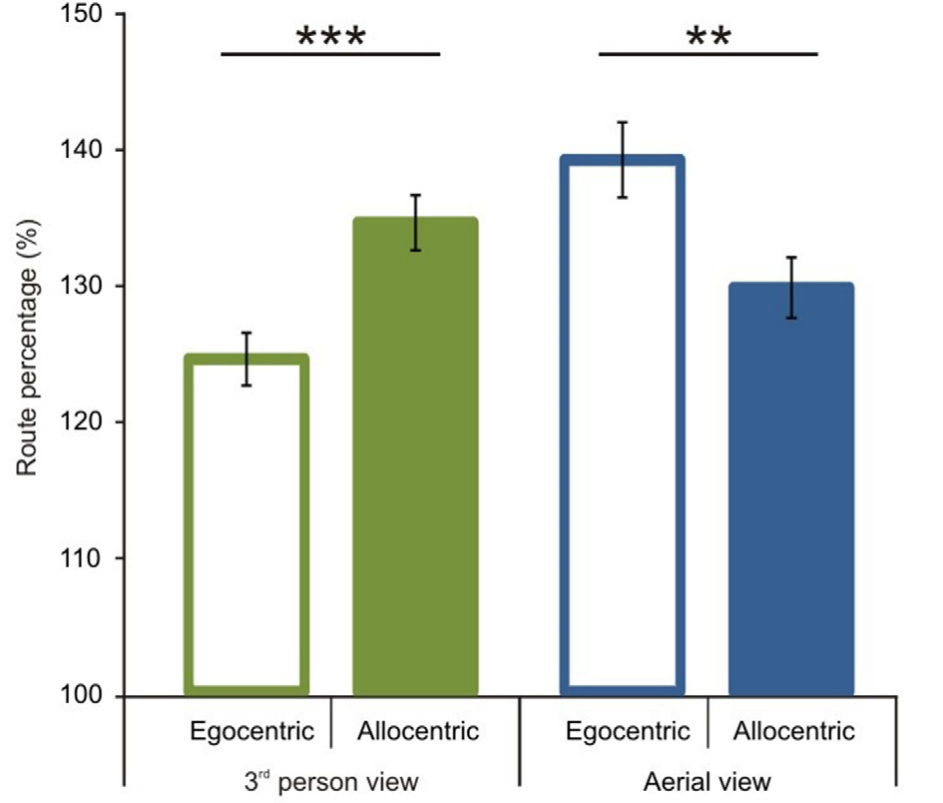
**Route performance scores according to viewing conditions and reference frames**. A significant interaction was found between point of view and frame of reference. In the 3rd person view egocentric frame of reference and in the aerial view allocentric frame of reference was preferred. Vertical bars denote standard errors. ^**^p < 0.01; ^***^p < 0.001.

We first analyzed route performance scores (see calculation in the Data Analyses section). We compared 1P-E and 3P-E viewing conditions to see whether a first person vs. third person point of view produced consistently different performance results (see Figure 3). A paired sample *t*-test showed no significant difference [*t*_(1, 49)_ = 0.2802, *p* = 0.7805, Confidence interval: 5.8079, −4.3867]. This suggests that the 3P-E point of view is no better or worse for virtual navigation than the natural 1st person, egocentric perspective. We followed by comparing route performance for the different viewing conditions in a 2 (*point of view*) by 2 (*frame of reference*) repeated measure mixed ANOVA, using Gender as a grouping variable. Results showed a main effect of point of view [*F*_(1, 48)_ = 8.472, *p* = 0.0055, η^2^_*p*_ = 0.1500] indicating that route lengths were closer to optimal from the ground-level (3P-E, 3P-A) than from aerial point of view (AE-A, AE-E) (see Figure 4). Furthermore, we found a strong interaction effect between *frame of reference* and *point of view* [*F*_(1, 48)_ = 34.178, *p* < 0.0001, η^2^_*p*_ = 0.4159]. *Post-hoc* comparison in a Tukey HSD test showed (*p* = 0.001) that 3P-A performance (*M* = 134.59, *SD* = 14.41) was inferior to 3P-E (*M* = 124.53, *SD* = 13.73) performance. Therefore, from the ground-level point of view, an egocentric frame of reference provided for better route length performance than an allocentric-frame of reference did. Meanwhile, the difference between AE-A (*M* = 129.80, *SD* = 15.80) and AE-E (*M* = 139.22, *SD* = 19.64) showed that from the aerial point of view, the allocentric frame of reference was preferred (*p* = 0.0020). The effect of gender on the interaction reached significance [*F*_(1, 48)_ = 4.445, *p* = 0.0402, η^2^_*p*_ = 0.0848], as female participants displayed a stronger *frame of reference* and *point of view* interaction.

### Optimality of time performance

**Figure 5:**
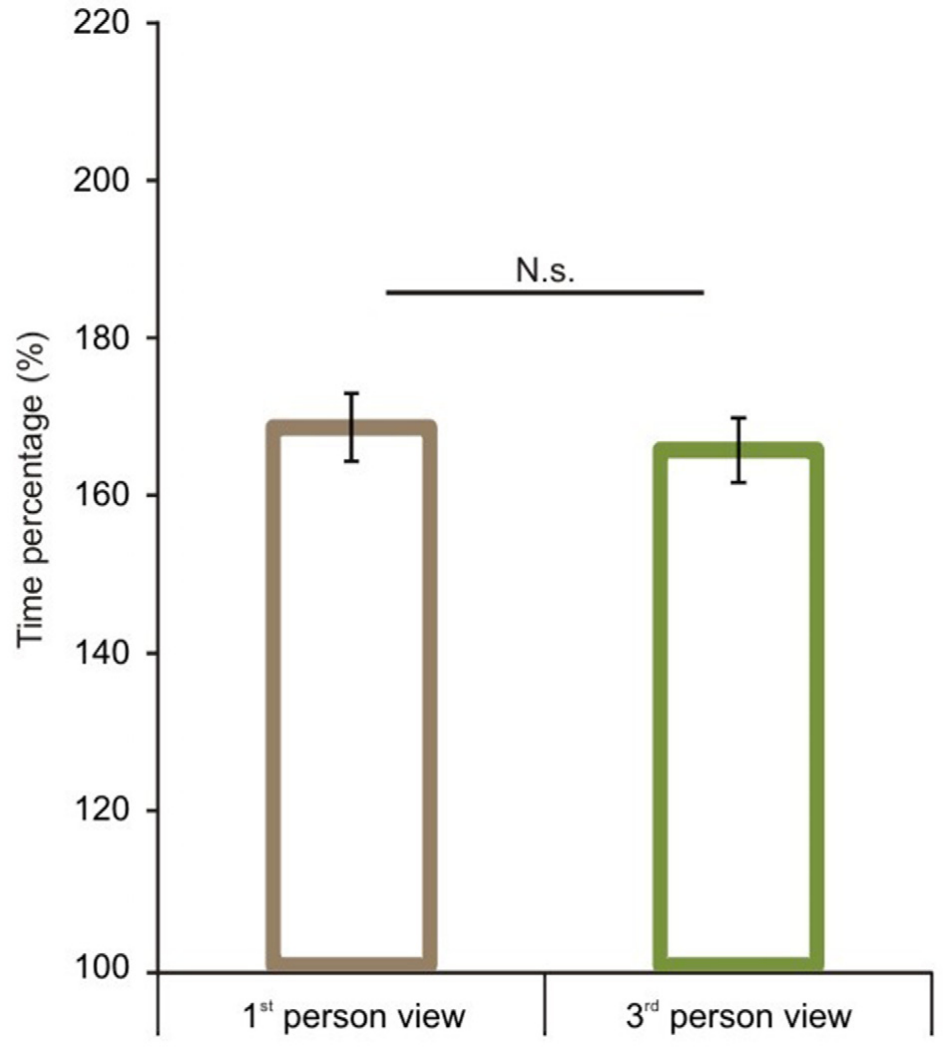
**Time performance scores in the 1st and 3rd person viewing conditions**. We found no significant difference between 1st person and 3rd person views when both share an egocentric frame of reference. Vertical bars denote standard errors. n.s., not significant.

**Figure 6:**
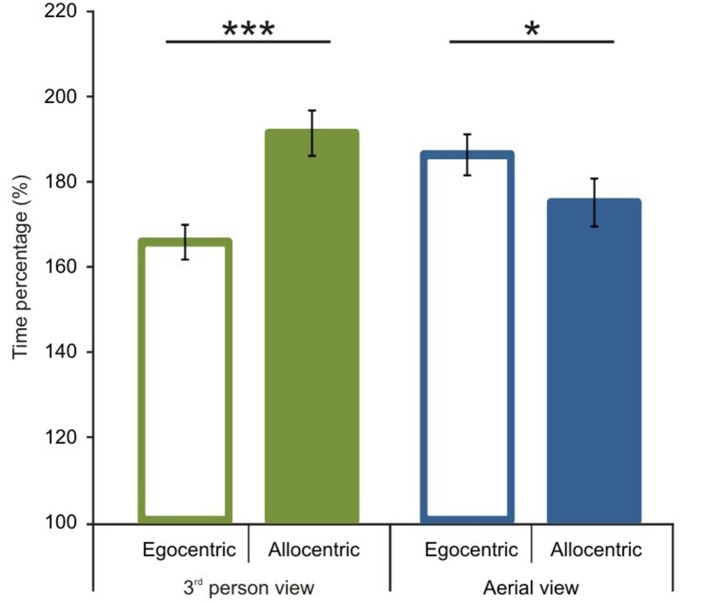
**Time performance scores according to viewing conditions and reference frames**. Significant interaction was found between point of view and frame of reference. In the 3rd person view, egocentric frame of reference was preferred. In the aerial view a preference was present for an allocentric frame of reference. Vertical bars denote standard errors. ^*^p < 0.05; ^***^p < 0.001.

After the comparison of route performance scores, we examined time performance scores (see calculation in the Data Analyses section). Starting with a comparison between 1P-E and 3P-E conditions, we found no significant difference [*t*_(1, 49)_ = 0.609, *p* = 0.5454, Confidence interval: 12.4416, −6.6551] (see Figure 5) as was found with the route length performance analysis. We then compared time performance scores in a 2 by 2 (*Point of view* by *Frame of reference*) repeated measure ANOVA using gender as the grouping variable. We found that male participants had better time percentage scores than women [*F*_(1, 48)_ = 4.873, *p* = 0.0321, η^2^_*p*_ = 0.0922]. Most importantly, results showed an interaction between *point of view* and *frame of reference* [*F*_(1, 48)_ = 48.221, *p* < 0.0001, η^2^_*p*_ = 0.5011; see Figure 6]. *Post-hoc* analyses of means by Tukey HSD test showed (*p* < 0.001) that 3P-A performance (*M* = 191.19, *SD* = 37.77) was again inferior to 3P-E performance (*M* = 165.54, *SD* = 29.08). This suggests that in the ground-level point of view, an egocentric frame of reference leads to faster route planning. *Post-hoc* test also showed (*p* = 0.022) that, again, AE-A performance (*M* = 174.84, *SD* = 39.82) was better than that of AE-E (*M* = 186.11, *SD* = 34.04). This provides further evidence that an allocentric frame of reference is preferred when using an aerial point of view the. Route performance was significantly faster (*p* = 0.029) in 3P-E than in the AE-A condition, but the AE-A condition was better than the 3P-A (*p* = 0.0005). The gender, point of view and frame of reference interaction did not reach significance.

In summary, we found that route performance was better overall when taking a ground-level point of view over an aerial view. Furthermore, we found an interaction between point of view and frame of reference, both regarding route- and time-performance scores. The interaction showed that from the ground perspective the egocentric frame of reference is preferred, while from the aerial perspective the allocentric frame of reference has an advantage. We found that men typically collected more aliens in the game than women, though this could be partly attributable to their overall faster route performance.

## Discussion

In the present study we examined the effect of viewpoint perspectives and frames of reference on performance in a virtual navigation task. We found that a ground level perspective led to better performance if it was associated with an egocentric, as opposed to allocentric, frame of reference. Meanwhile, when given an aerial point of view, the use of an allocentric frame of reference led to superior performance over an egocentric one. Overall, the ground-level/egocentric combination and the aerial-view/allocentric combination provided users with the best performance conditions, though the former was most superior. Our results also showed that men performed slightly better in general by collecting more targets in the game. This was partly attributable to men taking routes more time optimal than women, and because the interaction between frame of reference and point of view was stronger for women.

Our results are in line with earlier theories suggesting that ground level navigation activates egocentric frames of reference (Linde and Labov, 1975; Siegel and White, 1975). It also agrees with results on the use of orientation fixed maps lead to better performance (Aretz, 1991; McCormick et al., 1998). Earlier results showed that perspective and frame of reference both affect navigation performance, but to our knowledge this study provides the first direct evidence that an egocentric reference frame is more effective in ground-level navigation than allocentric and that an allocentric reference frame allows for more accurate navigation in map-like aerial perspectives. In contrast with earlier experiments where several landmarks were present within the visible area, the subjects in our experiment relied only on path integration with the help of environmental boundaries only.

We found that the navigation performance did not noticeably differ between first person and third person viewpoints. This observation has important implications for spatial cognition research. (1) Most studies to date have used a first person viewpoint for navigation experiments (e.g., Caplan et al., 2003; Ekstrom et al., 2003; Bird et al., 2010), because a third person point of view is thought to yield a less immersive experience, despite the player's self-projection into the body of the avatar (Slater et al., 2010). (2) Against this assumption, but consistent with other studies, spatially important aspects (distances) are just as accurately perceived from a third person point of view (Mohler et al., 2010; Lin et al., 2011). (3) Moreover, considering that VR navigation does not provide any proprioceptive cues that can be used to discriminate between the navigation with respect to the avatar from a 3rd-person view vs. first person point of view (Ruddle et al., 1998), it is plausible that the 3rd-person point of view does not conflict with the first person experience. Our results suggest that if the FOV is balanced between first person and third person viewpoints, then navigation performance does not differ either in route planning time or in route length. (4) Notably, many of our subjects were also accustomed to videogame experiences in which the player is represented by an avatar. Also note that cinematography has long been exploiting the capacity of the human brain to seamlessly perform projective transformations that allow for immersing ourselves into a protagonist's point of view. Whether this capacity is the result of learning or a product of natural cognitive development is a subject of future research.

The current behavioral results argue for the importance of manipulating these features when studying the neural circuitry of spatial navigation on different species and comparing results across species and virtual reality paradigms (Shelton and McNamara, 2004; Zaehle et al., 2007; Jacobs et al., 2013). During natural navigation, kinesthetic and visual input provides important references for computing heading and position (Ekstrom et al., 2003; Waller et al., 2008) as we continuously update our knowledge of the environment. This position updating involves the interaction of several brain areas. Linking our past viewpoint with current and future ones through path integration helps us to construct a route, which is a prerequisite of route knowledge. It is thought that at least two areas play an important role in viewpoint matching: the parahippocampal place area and the retrosplenial cortex (Park and Chun, 2009). The parahippocampal place area helps us in the discrimination of old and new viewpoints, while the retrosplenial cortex actively integrates viewpoints of the same environment (Wolbers and Büchel, 2005; Park and Chun, 2009). These and other results (Zhang et al., 2012) suggest that scene matching is an important part of navigation. The closer the successive viewpoints are, the easier it is to integrate them.

In their disorientation study, Waller and Hodgson (2006) found that subjects maintain egocentric localization in blindfolded pointing tasks after less than 135° of rotation, but switch to allocentric localization after larger rotations. This might explain our observation that ground level perspectives are associated with egocentric reference frame. From ground level perspectives, mental rotations are small so it is simple to match our 3rd person viewpoints with the avatar's. In contrast, an aerial perspective requires larger mental rotations with large potential errors, thus leaving the allocentric frame as a better option. The advantage gained by maintaining the egocentric transformations between ground-level perspectives appears to outweigh the ease of updating only one position in an allocentric frame as opposed to the whole scene in an egocentric frame (Burgess, 2006).

The finding that an aerial or out-of-environment perspective in large space navigation is associated with an allocentric frame of reference is in line with similar results from experiments in small spaces that could be manipulated (Burgess, 2006; Mou et al., 2006). Neuropsychological evidence provides further insights concerning the differences between ground level and map-like perspectives (Farrell, 1996; Takahashi et al., 1997). For example, Mendez and Cherrier (2003) described a patient with topographagnosia who, after a left occipitotemporal stroke (that affected the retrosplenial cortex), was unable to navigate in a familiar environment, but was able to draw and read maps. Such cases implicate that neural systems underlying ground level and map based navigation are partially independent. Moreover, representation of space (e.g., by drawing a map) and navigation in space might be performed by distinct neuronal computations (see also Zhang et al., 2012). In their study, Shelton and Gabrieli (2002) also found that participants followed different strategies in map drawing depending on previous ground level or aerial exploration. After ground level exploration they drew landmarks sequentially following their route, while after learning from an aerial perspective they drew the landmarks on the map consistent with a hierarchical strategy.

Probably the most important question derived from our study is to determine which feature of the camera's position caused the switch between ego- and allocentric reference frames. We can consider at least two explanations based on the differences between the aerial and 3rd person cameras used in the current study. One could argue that if the angular difference between the camera view and the avatar exceeds a given value then an allocentric reference frame is preferred as consistent with the above mentioned Waller and Hodgson finding (2006). It is also conceivable that simply the change in distance between the camera and the avatar may cause the switch itself. Further studies are necessary for addressing these questions, e.g., by systematically manipulating the distance or the angular difference between the camera and the avatar.

Our finding that an aerial point of view resulted in performance that was slightly inferior to ground-level performance could also be due to the enhanced visual details that ground level perspectives provided by the proximal environment. Also, the current task involved using egocentric controls (left, right) that may also bias performance in favor of egocentric navigation. Notably, in the current experiment the environment was square-shaped so the edge length provided no intrinsic cue of direction. Earlier studies showed that intrinsic axes in an environment play an important role in the preference of allocentric strategies (Mou et al., 2006, 2008).

Yet another factor may have also contributed to the difference between performance under ground-level views and aerial views in our experiment. Namely, the square environment provided a reliable geometry cue about the correct locations of the spaceships, even though the spaceships were not in the corners. While the walls were always visible from the third person point of view, neither orientation cues (sky, shadows), nor visible landmarks were available. It is a question whether the performance would have changed if the environmental borders were circular (or even invisible).

We found significant gender differences in performances as males overall earned more points in the task and also planned routes faster than women. This result is in line with earlier findings showing that males tend to rely on geometry and path integration, whereas women tend to rely more on landmarks (Chen et al., 2008; Andersen et al., 2012). However, one might argue that the use of a male avatar for both subject genders might have contributed to this result. While the argument has some validity, a study by Slater et al. (2010) showed that male participants were able to successfully project the body of a female avatar as theirs. The converse would be assumed as well. Moreover, none of the female participants considered the avatar's gender relevant enough to mention in debriefing.

The method used is also novel because, to our knowledge, it is the first implementation of a spatial navigation paradigm for an Android-based tablet PC. Participants were able to control their movements with a multi-touch screen. Although tablet PCs are not yet optimized for neuroscience research, they have an increasing potential for the adaptation of current paradigms. These devices provide a high-resolution display, powerful graphical rendering, are light-weight and are able to operate for up to 8 h on their built-in batteries. Relying on battery power is ideal for research because it does not generate AC artifacts and is easy to handle in clinical environments. We believe that multi-touch user interfaces, gesture control, and motion control through built-in webcam are viable alternatives for current keyboard control applications.

In conclusion, we found evidence for default associations between perspectives and frames of reference. First, we found that an egocentric frame of reference was preferred when the perspective was close to the eye level of the navigator and the transformation between our viewpoint and the avatar's was effortless. Second, we found that an allocentric frame of reference is preferred if the perspective is outside of the navigable area (in our case in the air) where viewpoint matching is hard but path integration relative to environmental cues was effortless. Furthermore, we found that first person and third person perspectives do not differ regarding navigation performance when the only difference is the presence or absence of an avatar in view. Lastly, we found that men performed better in our task. The significance of the current results is that they provide the first direct verification for the default frame of reference and point of view for spatial navigation.

### Conflict of interest statement

The authors declare that the research was conducted in the absence of any commercial or financial relationships that could be construed as a potential conflict of interest.
